# Congenital Granular Cell Tumor: Case Report and Review

**DOI:** 10.1155/2018/4389158

**Published:** 2018-09-26

**Authors:** Preston Gardner, Arlene Rozzelle

**Affiliations:** ^1^Department of Plastic & Reconstructive Surgery, Beaumont Hospital-Farmington Hills, 28050 Grand River Ave., Farmington Hills, MI 48336, USA; ^2^Department of Plastic & Reconstructive Surgery, Childrens Hospital of Michigan, Detroit Medical Center, 3901 Beaubien St., Detroit, MI 48201, USA

## Abstract

Congenital granular cell tumors are infrequently occurring masses occurring on a neonate's gingiva/alveolus. These lesions are benign with no noted malignant transformation, and treatment of excision is based on its effect on the neonate's respiratory ability and/or nutritional intake. The purpose of this review is to discuss a case of a congenital granular cell tumor and its treatment and review of the literature including demographics, histopathology, and operative treatment.

## 1. Introduction

Originally described by German pathologist, Ernst Christian Neumann in 1871 [1], congenital granular cell tumor (CGCT) has also been referred to as congenital epulis and Neumann tumor. The term epulis, derived from the Greek translation, meaning “on the gum,” being a benign proliferation on alveolar mucosa in a neonate. Since its initial report, as of 2002, there had been 216 documented cases, ranging in size from mere millimeters to upwards of 9 cm. As these lesions tend not increase in size and have been reported to regress without therapy, surgical excision is often deferred unless respiratory or feeding difficulty ensues [2].

## 2. Case Report

**Figure 1:**
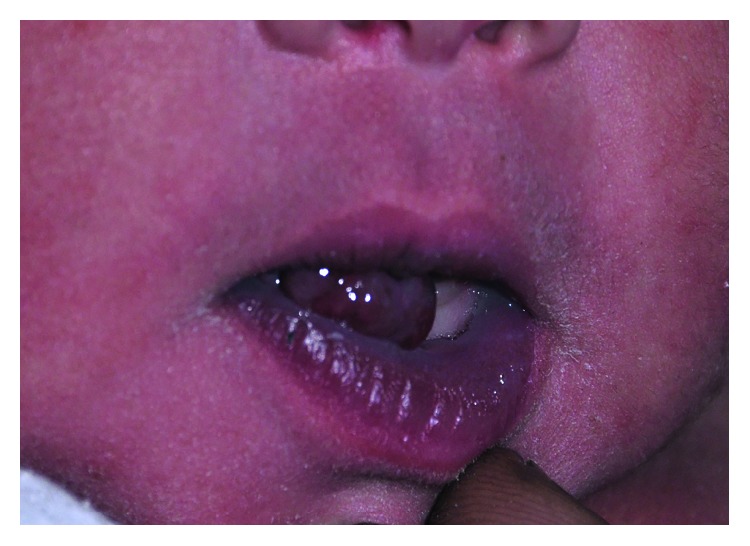
Patient as seen at initial consultation.

The patient is an African-American female born at 37 weeks six days gestation to a 19-year-old mother. She was noted to have a 1.5 cm pedunculated soft tissue mass with adjacent secondary 8 mm mass on the oral mucosa along the mandibular alveolar ridge (Figure 1).

**Figure 2:**
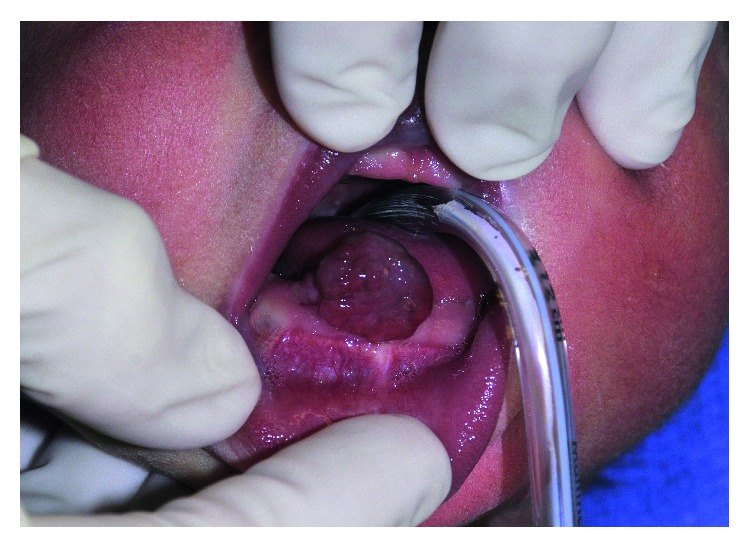
Patient in OR after intubation.

Due to difficulty with breast-feeding, decision was made for operative excision on the third day of life. In the operating room, she received general anesthesia with oral endotracheal intubation (Figure 2).

**Figure 3:**
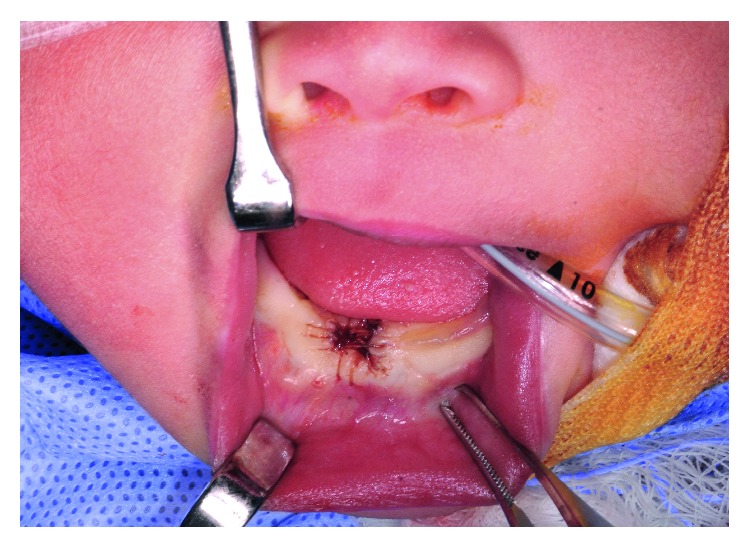
Wound following closure.

Local anesthetic of 0.6 cc 0.5% lidocaine with 1 : 200,000 epinephrine was administered at the base of the masses. Excision was performed sharply with scalpel and hemostasis with bipolar electrocautery. Complete closure of the defect was not possible, but was reapproximated to near closure with 5-0 chromic sutures in simple interrupted fashion in a crosshatched pattern (Figure 3).

**Figure 4:**
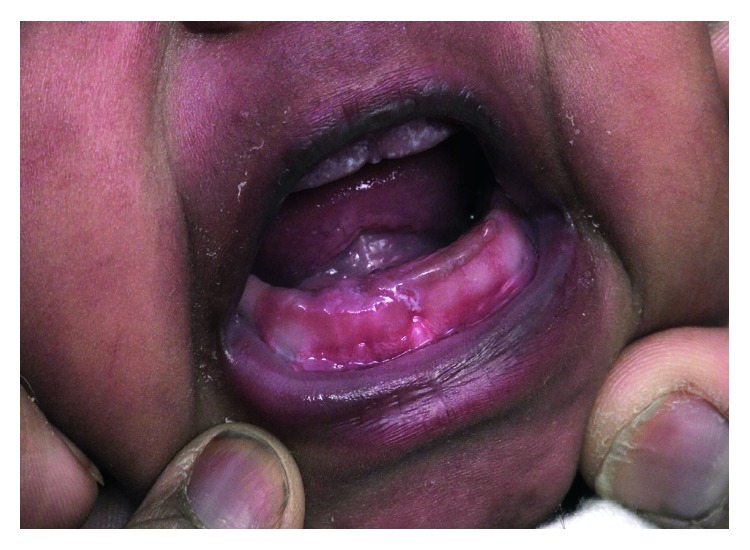
Patient at follow-up.

She was allowed to return to breast and formula oral intake postoperatively on the same day. She was monitored for two days and then discharged home. Follow-up after three weeks revealed well-healed mucosa at the surgical site with minimal notching on the alveolar ridge and no evidence of recurrence (Figure 4).

Microscopic pathology confirmed squamous mucosa with underlying large polyhedral cells containing granular acidophilic cytoplasm as well as small hyperchromatic nuclei, staining negative for S-100 immunohistochemistry, with extension to the excisional base of the lesion. Centrally dilated blood vessels were seen with mild nonspecific chronic inflammatory changes as well as prominent nucleoli in some cells. No dysplasia or malignancy was noted centrally or peripherally. All microscopic findings appear consistent with that of CGCT.

## 3. Discussion

Due to the infrequency of CGCT occurrence, it has mostly been noted in the literature via case reports and literature reviews. Two of the larger works include that of Dash et al. including fifty patient reviews and Lack et al. including 21 patient reviews noting multiple commonalities. There is a tendency for CGCT to develop on the alveolar ridge, particularly that of the maxilla with a threefold predilection over that of the mandible, particularly in the area of canine and lateral incisors, theorized to be secondary to common local occurrences of supernumerary teeth. CGCT presents as a solitary lesion 90% of cases. A female predisposition accounts for eightfold increased incidence above that of males, with no currently known reason, as CGCT has not been found to contain estrogen nor progesterone hormonal receptors [3, 4]. There is a noted higher incidence in the Caucasian population [5].

Grossly, CGCT appears well developed in the newborn as a variably sized soft tissue mass, with a tan, pink, or red coloration, and an irregular, lobulated, and/or smooth surface, typically arising from the alveolar ridge [2, 6, 7]. Much emphasis of study has been made on pathological evaluation. Microscopic characteristics include hypervascularity, large granular cells with significant eosinophilic cytoplasm, and small basophilic nuclei. A multitude of immunoreactive studies may be performed including S-100, CD34, CD68, CD105, and many others. S-100 remains one of the most important immunohistochemical evaluations, particularly as adult granular cell tumor, and CGCT may grossly be difficult to distinguish, granted with different clinical presentations of patient age, staining positive in adult form and negative in CGCT [6]. While CGCT tends to be diagnosed upon delivery, documentation in the literature has shown diagnosis of larger lesions on prenatal ultrasonography during the third trimester as well as associated polyhydramnios secondary to poor swallowing en utero [5]. Further workup of patients with CGCT has been performed with extensive blood analysis, karyotype, and X-ray/computed tomography imaging, without noted anomaly or associated malformations [5, 8].

Generalized treatment of CGCT remains debated, unless the newborn patient has respiratory compromise or nutritional intake impairment secondary to the mass causing oral obstruction. If the lesion is small without obstructive signs/symptoms, some propose a conservative approach, demonstrated by Jenkins and Hill in their patient with a 1.5 cm mass, regressing to 3 mm at 12 month follow-up. With obstructive issues, general consensus is simple excision [9]. Others have advocated alternate therapy such as CO2 laser excision with successful outcome [10]. Depending on the size of the CGCT, if discovered via prenatal ultrasound, ex utero intrapartum treatment has been proposed if there is high risk for respiratory distress/upper airway obstruction [5]. Some discussion has been debated as to modality of defect closure following lesion excision. Following excision with CO_2_ laser by Lapid et al., the wound was not closed, allowed to heal by secondary intention, with gingiva reepithelialization noted at ten-day follow-up. There was no discussion as to long-term follow-up, particularly in regard to subsequent dental development [10]. Narasimhan et al. however advocate a gingivoperiosteoplasty, in their patient who coincidentally had an alveolar notch, similar to that of alveolar cleft management. Their rationale being appropriate dental development requires alveolar continuity; however, they noted only one documented case with resultant absence of local tooth development. At eighteen-month follow-up, their patient did have normal dental development [7]. Regardless of therapy, no reports have shown recurrence, malignant conversion, nor metastasis, and Lack et al. demonstrate no recurrence in fifteen patients at fifteen-year follow-up, eleven of which had incomplete excisions [4, 8, 9].
